# Comparative Transcriptomics Reveals Potential Regulatory Mechanisms Underlying Changes in Antioxidant, Anti-Inflammatory, and Other Immune Functions Induced by *Bellamya purificata* Polysaccharides in *Cherax quadricarinatus*

**DOI:** 10.3390/antiox15070907

**Published:** 2026-07-22

**Authors:** Chang Yuan, Huizan Yang, Xianhui Pan, Yong Lin, Kangqi Zhou, Feng Lin, Wenjian Chen, Yusen Li, Yaoquan Han, Hui Wei, Caiguang Wang, Liangliang Huang, Dapeng Wang

**Affiliations:** 1 College of Environmental Scince and Engineering, Guilin University of Technology, Guilin 541006, China; yc545372450@foxmail.com (C.Y.);; 2 Key Laboratory of Aquaculture Genetic and Breeding and Healthy Aquaculture of Guangxi, Guangxi Academy of Fishery Sciences, Nanning 530021, China

**Keywords:** *Cherax quadricarinatus*, *Bellamya purificata* polysaccharide, physiological stress, antioxidant capacity, antibacterial activity, anti-inflammatory function, transcriptome

## Abstract

This study investigated the potential effects of Bellamya purificata polysaccharide (BPP) supplementation in feed on *Cherax quadricarinatus*, aiming to elucidate its impact on immune function and the underlying regulatory mechanisms. A 60-day feeding trial was conducted to evaluate the effects of varying dietary BPP concentrations (0‰, 2‰, 4‰, 6‰, and 8‰) on growth performance, histopathology, immune function, and transcriptomic profiles of *C. quadricarinatus*. Dietary BPP significantly increased the final body length and body weight of *C. quadricarinatus*. Furthermore, BPP inclusion improved the histological damage of hepatopancreatic tissues, alleviated physiological stress, and enhanced antioxidant capacity, antibacterial activity, and anti-inflammatory function, as evidenced by alterations in relevant enzyme activities and gene expression levels. Transcriptomic analysis revealed 957 differentially expressed genes (DEGs), which were predominantly enriched in GO terms and KEGG pathways associated with BPP-mediated immune regulation, such as the PPAR signaling pathway, peroxisome, steroid hormone biosynthesis, and lysosome. In addition, 14 candidate genes were identified, including *Acox3*, *ACOX1*, *SCP2*, and *PPT1*. Integrated analysis of GO, KEGG, and Short Time-Series Expression Miner (STEM) data supported the development of a mechanistic model illustrating how *C. quadricarinatus* modulates immune function in response to BPP supplementation. RT-qPCR validation confirmed the accuracy and reliability of the high-throughput sequencing results. Collectively, these findings highlight the potential of BPP as a functional feed additive in aquaculture and provide novel insights into disease prevention strategies for the *C. quadricarinatus* farming industry.

## 1. Introduction

*Cherax quadricarinatus*, commonly referred to as the Australian freshwater blue crayfish, is in high market demand owing to its large body size, rapid growth rate, and high productivity [1]. At present, this species is extensively cultivated in Chinese provinces, including Guangdong, Guangxi, and Hainan [2]. The rapid expansion of aquaculture operations has resulted in increased stocking densities, which may adversely affect the immune system and elevate disease incidence [3]. To control bacterial, fungal, and viral infections, farmers often administer chemical agents and antibiotics during production [4]. Nevertheless, prolonged application of such substances can induce drug resistance in aquatic animals, disturb immune homeostasis, and contribute to environmental pollution [5]. Consequently, the identification of suitable alternatives and the development of safe, efficacious immunomodulators—while minimizing reliance on chemical drugs and antibiotics—hold considerable practical value for sustainable aquaculture.

Polysaccharides, a diverse class of naturally occurring biopolymers, have attracted extensive attention as promising immunostimulatory feed additives in aquaculture [6]. A comprehensive review has recently concluded that polysaccharides can stimulate immune responses, promote growth, and enhance gut health in both fish and crustaceans, positioning these natural compounds as preventive, eco-friendly, and effective functional feed ingredients [7]. Within the broader context of polysaccharide research, polysaccharides extracted from mollusks (particularly freshwater gastropods) represent a relatively underexplored but potentially valuable resource [8]. Evidence from glycobiology research has established that mollusk-derived polysaccharides possess a diverse range of bioactivities, including anti-tumor, antiviral, anticoagulant, hypolipidemic, hypoglycemic, and pronounced immunomodulatory properties [9,10,11]. The freshwater snail *Bellamya purificata*, which is widely distributed in the Yangtze River basin and southern China, is a gastropod mollusk of the family Viviparidae that has attracted particular research interest [12]. Widely consumed as an aquatic food in China, *B. purificata* has long been recognized in traditional Chinese medicine for its pharmacological properties and is traditionally used in the treatment of dysentery, hemorrhoids, jaundice, and eczema [13]. Critically, previous studies have preliminarily demonstrated that the polysaccharides BSP-1 and BSP-2, isolated from the muscle tissue of *B. purificata*, effectively inhibit the production of nitric oxide and proinflammatory cytokines in both in vitro and in vivo models, suggesting that *B. purificata* polysaccharides hold considerable potential as natural anti-inflammatory and immunomodulatory agents [13,14]. Furthermore, our team has successfully isolated a homogeneous *B. purificata* polysaccharide (α-D-glucan) and validated its lipid-lowering and hepatoprotective effects in a high-fat-diet zebrafish model [15]. Although the biological activity of *B. purificata* polysaccharides has been demonstrated in cellular and animal model systems, their immunomodulatory effects in *C. quadricarinatus* remain entirely unexplored. Transcriptomic methods have been widely applied to *C. quadricarinatus* and other crustaceans [16]. However, to date, no studies have been reported that use transcriptomic methods to investigate the immunomodulatory effects of *B. purificata* polysaccharides in *C. quadricarinatus*. Understanding how *B. purificata* polysaccharides confer hepatoprotective effects and engage the conserved innate immune pathways in this economically important crustacean species would not only address a fundamental scientific question but also provide a molecular framework for developing functional hepatopancreas-targeted immunostimulants to achieve sustainable disease management in crayfish aquaculture.

Comparative transcriptomics has emerged as a powerful and unbiased approach for elucidating the transcriptional changes underlying complex physiological processes, including immune activation and regulation [17]. Advances in next-generation sequencing (NGS) technologies facilitate comprehensive profiling of gene expression across diverse tissues, experimental conditions, and developmental time points, thereby offering profound insights into gene regulatory networks and organismal biology [18,19,20]. In recent years, transcriptomic approaches have been increasingly employed to investigate the immunomodulatory effects of various polysaccharide extracts in crustaceans, including *Litopenaeus vannamei* [21], *Procambarus clarkii* [22], and *Eriocheir sinensis* [23].

To this end, the present study evaluated the effects of dietary inclusion of five different BPP concentrations (0‰, 2‰, 4‰, 6‰, and 8‰) on *C. quadricarinatus*, with particular emphasis on hepatopancreatic histology and immune function, encompassing physiological stress responses, antioxidant capacity, antibacterial activity, and anti-inflammatory competence. In addition, transcriptomic profiling of hepatopancreatic tissue was performed to elucidate the underlying molecular regulatory mechanisms. The results provide fundamental insights into the potential application of BPP as an immunomodulator for ameliorating hepatopancreatic injury and enhancing overall health in *C. quadricarinatus* and establish a scientific foundation for the formulation of novel disease prevention strategies in *C. quadricarinatus* aquaculture.

## 2. Materials and Methods

### 2.1. BPP and Dietary Preparation

*Bellamy a purificata* polysaccharide (BPP) was extracted by Jiangsu Sanshu Biotechnology Co., Ltd. (Nantong, China). BPP consisted primarily of a homogeneous neutral polysaccharide identified as α-D-glucan, with a molecular weight (Mw) of 6412.704 kDa and 97% purity. All feed ingredients were of animal feed grade and obtained from Guangdong Hengxing Feed Industry Co., Ltd. (Zhanjiang, China). Experimental diets with varying BPP concentrations were formulated following the protocol described by Zhang et al. [24]. Specific quantities of BPP powder were incorporated into the basal diet and homogeneously blended to achieve final concentrations of 0‰, 2‰, 4‰, 6‰, and 8‰. The feed preparation procedure was as follows: The basal diet was pulverized to pass through a 60-mesh sieve using a hammer mill. Predetermined amounts of BPP were added to the ground basal diet according to the target concentrations. Each mixture was blended uniformly for 15 min using a drum mixer. Sterile distilled water was added to each mixture at a ratio of 40% (*w*/*w*) to form a dough-like consistency. The dough-like mixture was extruded through a pellet mill to produce pellets with a diameter of 1–1.5 mm. Pellets were air-dried at 30 °C until the moisture content was reduced to less than 10 g per 100 g. Dried pellets were sealed in plastic bags and stored at −20 °C until use. Fresh batches of feed were prepared weekly. The detailed formulation of the experimental diets is provided in Table S1.

### 2.2. Experimental Design and Sample Collection

The *C. quadricarinatus* used in this study were obtained from the breeding facility of the Guangxi Academy of Fisheries Sciences. Prior to the experiment, the crayfish were temporarily held in aquaria under controlled environmental conditions: water temperature 26–28 °C, pH 7.6–8.0, and dissolved oxygen concentration > 5 mg/L. Following a 7-day acclimation period, a 60-day feeding trial was initiated using healthy individuals. A total of 450 crayfish (mean weight 0.18 ± 0.04 g; mean body length 1.75 ± 0.38 cm) were randomly allocated to 15 rearing tanks (1 × 2 × 1 m), with 30 individuals per tank. The crayfish in these 15 rearing tanks were divided into 5 groups (control group: 0‰; BPP treatment groups: 2‰, 4‰, 6‰, and 8‰), with three replicates per group. PVC pipes were provided in each tank as shelters. Survival was assessed on day 60. At the conclusion of the experiment, all crayfish were fasted for 24 h prior to measurement of body length and weight. Throughout the trial, crayfish were fed twice daily (9:00 and 17:00) at a concentration of approximately 5% body weight, with adjustments made according to observed feeding intensity. Sampling was conducted after the fasting period. From each replicate, 12 individuals were randomly selected, and hepatopancreas tissues were dissected. The hepatopancreas tissue from one crayfish was prepared as a separate sample, fixed in 4% paraformaldehyde, and used for histopathological examination; the hepatopancreatic tissues from 6 crayfish were combined into a single sample, flash-frozen in liquid nitrogen, and used for enzymatic assays; the hepatopancreatic tissues from 5 crayfish were combined into a single sample, flash-frozen in liquid nitrogen, and used for transcriptomic sequencing and gene expression analysis.

Survival Rate (%) = Number of Survivors at End of Cultivation/Number of Survivors at Start of Cultivation × 100%.

### 2.3. Tissue Sections

The preparation of hepatopancreas tissue sections was performed according to the method described by Yuan et al. [25]. In brief, hepatopancreatic tissues were fixed in 4% paraformaldehyde for 24 h. Subsequently, the samples were dehydrated through a graded ethanol series (75–95% ethanol) followed by absolute ethanol, cleared in xylene, embedded in paraffin, and sectioned at a thickness of 3–4 μm; sections were floated on a warm water bath to facilitate flattening. The sections were mounted onto glass slides and coverslipped. Hematoxylin and eosin (H&E) staining was then applied. Stained sections were examined under a light microscope (MSHOT, Guangzhou, China) at 100× magnification, and digital images were acquired for analysis. The MSHOT image analysis system (version 1.1.4) was employed to determine the average area of blister cells and resorptive cells.

### 2.4. Indicators Related to Immune Function

Enzymatic activities of seven immune-related enzymes were determined according to the manufacturer’s instructions. Assays were performed using commercial kits (Nanjing, China) supplied by Nanjing Jiancheng Institute of Biological Engineering to quantify catalase (CAT, U/g), superoxide dismutase (SOD, U/g), glutathione peroxidase (GSH-Px, mU/g), malondialdehyde (MDA, nmol/g), acid phosphatase (ACP, U/g), alkaline phosphatase (AKP, U/g), and lysozyme (LYS, U/L).

Gene expression levels of nine immune function-associated genes were analyzed using reverse transcription quantitative PCR (RT-qPCR). Total RNA was extracted from hepatopancreas samples using TRIzol reagent, and cDNA was synthesized using the Novozymes Reverse Transcription Kit (R223). β-Actin was selected as the internal reference gene. Primer sequences for activating transcription factor (*ATF*), crustin, prophenoloxidase (*proPO*), phenoloxidase (*PO*), heat shock protein 70 (*hsp70*), heat shock protein 90 (*hsp90*), C-type lectin (*CTLs*), cysteine protease 2 (*Cpa2*), and Toll-like receptor 13 (*TLR13*) are listed in Table S2. The RT-qPCR reaction mixture (20 μL) comprised 10 μL of 2× ChamQ SYBR qPCR Master Mix (Novozymes), 0.4 μL each of forward and reverse primers (10 μM), 4 μL of cDNA template, and 5.2 μL of deionized water. Amplification was performed on an ABI StepOnePlus Real-Time PCR System (Thermo Fisher Scientific, Waltham, MA, USA) under the following cycling conditions: initial denaturation at 95 °C for 90 s, followed by 40 cycles of 95 °C for 5 s, 60 °C for 15 s, and 72 °C for 20 s. Relative gene expression was calculated using the 2^−ΔΔCt^ method [26]. All RT-qPCR reactions were conducted in triplicate. Expression data are presented as mean ± standard deviation.

ACP, AKP, *hsp70*, and *hsp90* were assessed as indicators of physiological stress [25,27,28]. CAT, GSH-Px, SOD, and MDA were evaluated to reflect antioxidant capacity [29,30,31,32,33]. LYS, *ALF*, crustin, *proPO*, and *PO* served as markers of antibacterial activity [34,35,36,37]. *CTLs*, *Cpa2*, and *TLR13* were measured to indicate anti-inflammatory capacity [38,39,40,41].

### 2.5. RNA Extraction, cDNA Library Construction, and Illumina Sequencing

Total RNA was extracted from individual hepatopancreas samples using the TRIzol reagent kit (Invitrogen, Shanghai, China). RNA integrity and the absence of genomic DNA contamination were verified by agarose gel electrophoresis. For cDNA library construction, the following procedures were carried out using the mRNA Library Preparation Kit (New England Biolabs, Ipswich, MA, USA): mRNA isolation and fragmentation, double-stranded cDNA synthesis and purification, end repair, polyadenylation and adapter ligation compatible with Illumina sequencing platforms, size selection of approximately 200 bp cDNA fragments, and PCR amplification followed by purification. All cDNA libraries were sequenced on an Illumina HiSeq™ 4000 system.

### 2.6. Transcriptome Assembly

Raw sequencing reads were subjected to quality filtering using Fastp software [42]. Specifically, reads containing adapter sequences, those with >10% unknown nucleotides, and those with >50% low-quality bases (Q ≤ 20) were removed. The filtered high-quality reads were aligned to the *C. quadricarinatus* reference genome (NCBI accession: GCF_026875155.1) using HISAT2 [43]. Transcript reconstruction was performed with StringTie [44] to identify genes detected in the current sequencing run but absent from the reference genome (or reference gene annotation set); these were defined as novel genes. Based on the total number of genes annotated in the reference genome, the numbers of known and novel genes detected in the sequencing data were enumerated to evaluate sequencing quality and the completeness of the reference genome. A low detection rate of known genes may indicate inadequate sequencing depth or coverage, whereas a high number or proportion of novel genes may suggest that the reference genome is incomplete.

### 2.7. Analysis of the Identification and Functional Characterization of Differentially Expressed Genes

Gene expression levels for all samples were quantified using RSEM [45] and expressed as fragments per kilobase of transcript per million mapped reads (FPKM). By calculating the Pearson correlation coefficient between any two samples based on their expression levels, we can assess the quality of replication among replicate samples within a group. Differential gene expression analysis between sample groups was performed using DESeq2 [46], with raw read count data serving as input. Briefly, following normalization of read counts, *p*-values were computed based on the statistical model, and multiple-testing correction was applied to derive false discovery rates (FDR). Differentially expressed genes (DEGs) were identified using the following criteria: FDR < 0.05 and |log_2_(fold change)| > 1.

Potential biological functions of the DEGs were investigated through Gene Ontology (GO) and Kyoto Encyclopedia of Genes and Genomes (KEGG) pathway enrichment analyses, as well as Short Time-Series Expression Miner (STEM) analysis. Enrichment annotations were retrieved from the GO database (http://www.geneontology.org/, accessed on 10 November 2025) and the KEGG database (https://www.kegg.jp/, accessed on 10 November 2025), respectively, with a Q-value ≤ 0.05 considered statistically significant. Temporal expression patterns of DEGs were analyzed using the STEM software [47]. Nine DEGs were selected for RT-qPCR validation, with β-actin as the internal reference gene; primer sequences are provided in Table S2. The RT-qPCR procedures were identical to those described in Section 2.4.

### 2.8. Data Statistics

One-way analysis of variance (ANOVA) was performed using SPSS 20.0 software. Data are presented as “Mean ± standard deviation,” and a *p* < 0.05 indicates statistical significance.

## 3. Results

### 3.1. Effects of BPP in the Diet on the Growth Performance of C. quadricarinatus

**Figure 1:**
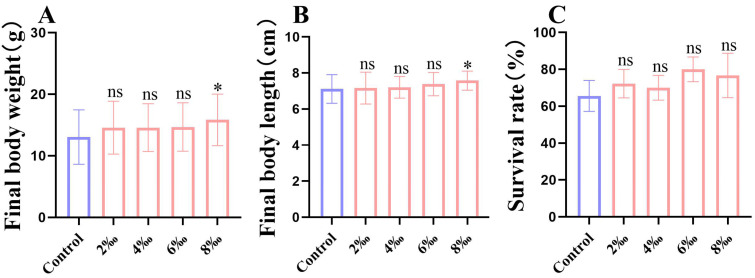
Effects of BPP on the growth performance of *Cherax quadricarinatus*. (**A**) The effect of BPP on the final body weight of *C. quadricarinatus*; (**B**) The effect of BPP on the final body length of *C. quadricarinatus*; (**C**) The effect of BPP on the survival of *C. quadricarinatus*. Compared with the control group, * indicates *p* < 0.05 and ns indicates *p* > 0.05 (no significant difference) in the BPP-treated group.

Figure 1 illustrates changes in the growth performance of *C. quadricarinatus* following dietary administration of different BPP concentrations. No statistically significant differences were observed in final body weight or body length between the control group (13.04 g and 7.11 cm) and the 2‰ (14.56 g and 7.16 cm), 4‰ (14.57 g and 7.21 cm), or 6‰ (14.67 g and 7.38 cm) treatment groups (*p* > 0.05) (Figure 1A,B). In contrast, both final body weight and final body length were significantly greater in the 8‰ treatment group (15.84 g and 7.58 cm) compared with the control (*p* < 0.05) (Figure 1A,B). Survival rates did not differ significantly among the control group (65.56%) and the 2‰ (72.22%), 4‰ (70.00%), 6‰ (80.00%), and 8‰ (76.67%) treatment groups. These findings indicate that dietary supplementation with high concentrations (8‰) of BPP enhances growth performance in *C. quadricarinatus*.

### 3.2. Effects of BPP Feeding on the Histopathology of the Hepatopancreas in C. quadricarinatus

**Figure 2:**
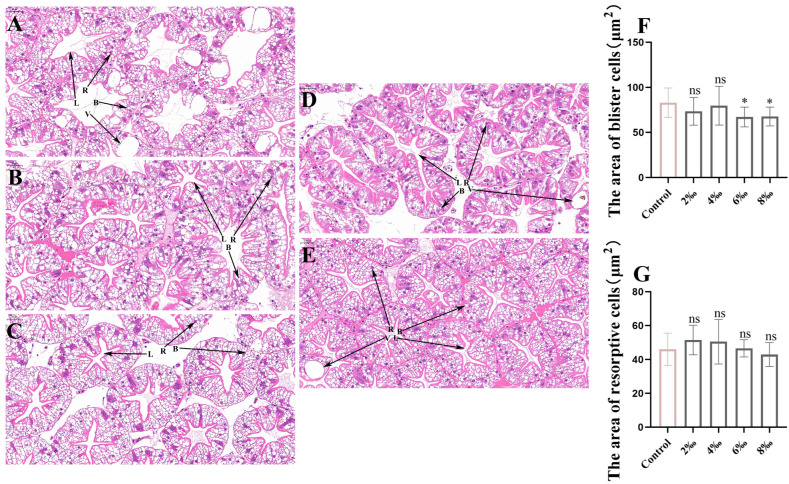
Effects of BPP on hepatopancreatic histopathology in *Cherax quadricarinatus*. (**A**–**E**) Representative photomicrographs of hepatopancreas sections from *C. quadricarinatus* fed diets supplemented with BPP at concentrations of 0‰, 2‰, 4‰, 6‰, and 8‰. L, lumen; B, blister cells; R, resorptive cells; V, vacuolation. (**F**,**G**) Variations in the average area of blister cells and resorptive cells across the different BPP treatment groups. Compared with the control group, * indicates *p* < 0.05 and ns indicates *p* > 0.05 (no significant difference) in the BPP-treated group.

To investigate the effects of BPP on the hepatopancreatic histopathology of *C. quadricarinatus*, the structural morphology of the hepatopancreas was examined microscopically. In the control group, hepatopancreatic tissue appeared compact and structurally intact, with distinct basal membrane boundaries, clearly defined stellate lumina, and evenly distributed blister cells and resorptive cells. However, a minority of blister cells exhibited cytoplasmic swelling and vacuolation, and certain lumina appeared slightly loosened with increased intercellular spacing (Figure 2A). In contrast, hepatopancreatic tissues from the treatment groups (2‰, 4‰, 6‰, and 8‰ BPP) displayed varying degrees of structural improvement (Figure 2B–G). Notably, stellate lumina became more regular and compact, the average area of blister cells decreased, and instances of abnormal swelling and vacuolation were reduced (Figure 2B–G). The most pronounced morphological improvement was observed in the 8‰ BPP group (Figure 2B–G). Although vacuolation persisted to some extent, the overall architecture of the hepatopancreas was more regular and orderly. Collectively, these findings indicate that dietary BPP alleviated mild hepatopancreatic damage in *C. quadricarinatus* in a concentration-dependent manner. Subsequently, the effects of BPP on immune function were evaluated, focusing on alterations in immune parameters and underlying regulatory mechanisms.

### 3.3. Effects of BPP in the Diet on Immune Function Parameters in the Hepatopancreas of C. quadricarinatus

**Figure 3:**
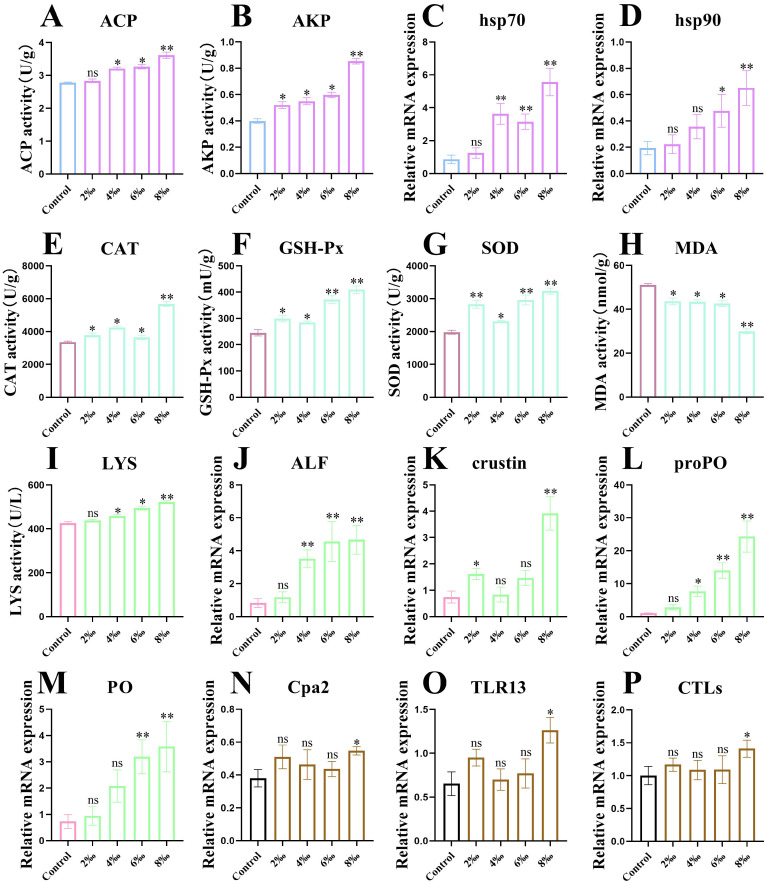
Effects of BPP on enzyme activities and immune-related gene expression in *Cherax quadricarinatus*. (**A**–**D**) Enzyme activities and gene expression associated with physiological stress responses. (**E**–**H**) Enzyme activities related to antioxidant capacity. (**I**–**M**) Enzyme activities and gene expression associated with antimicrobial activity. (**N**–**P**) Gene expression associated with anti-inflammatory capacity. Data are presented as mean ± standard deviation (SD) from three biological replicates. Statistical significance is indicated as follows: * indicates *p* < 0.05 and ** indicates *p* < 0.01; ns, not significant (*p* > 0.05).

To investigate the effects of BPP on the immune function of *C. quadricarinatus*, the activities of immune-related enzymes were measured in crayfish exposed to different dietary BPP concentrations. ACP activity in the 4‰ (3.21 U/g), 6‰ (3.27 U/g), and 8‰ (3.61 U/g) treatment groups was significantly higher than that in the control group (2.78 U/g), whereas no significant difference was observed between the 2‰ group (2.84 U/g) and the control (*p* < 0.05) (Figure 3A).

Activities of AKP, CAT, SOD, and GSH-Px in the 2‰ (0.52 U/g, 3755.34 U/g, 299.47 mU/g, and 2831.99 U/g), 4‰ (0.55 U/g, 4243.88 U/g, 284.69 mU/g, and 2314.03 U/g), 6‰ (0.60 U/g, 3648.51 U/g, 371.64 mU/g, and 2962.52 U/g), and 8‰ (0.85 U/g, 5673.44 U/g, 408.96 mU/g, and 3233.34 U/g) groups were significantly higher than those in the control group (0.40 U/g, 3359.53 U/g, 245.32 mU/g, and 1978.52 U/g) (*p* < 0.05) (Figure 3B,E–G). MDA levels in the control group (51.05 nmol/g) were significantly higher than those in the 2‰ (43.58 nmol/g), 4‰ (43.43 nmol/g), 6‰ (42.60 nmol/g), and 8‰ (29.91 nmol/g) treatment groups (*p* < 0.05) (Figure 3H). LYS activity in the control group (426.08 U/L) did not differ significantly from that in the 2‰ group (437.63 U/L) but was significantly lower than those in the 4‰ (458.43 U/L), 6‰ (494.48 U/L), and 8‰ (521.01 U/L) groups (*p* < 0.05) (Figure 3I). To further elucidate the immunomodulatory effects of BPP, the expression levels of immune-related genes were examined across the different treatment groups. mRNA levels of *hsp70*, *ALF*, and *proPO* in the control group (0.86, 0.82, and 1.04) did not differ significantly from those in the 2‰ group (1.25, 1.18, and 2.82) but were significantly lower than those in the 4‰ (3.64, 3.53, and 7.69), 6‰ (3.15, 4.56, and 14.03), and 8‰ (5.57, 4.67, and 24.31) groups (*p* < 0.05) (Figure 3C,J,L). mRNA levels of *hsp90* and *PO* in the 6‰ (0.48 and 3.20) and 8‰ (0.65 and 3.58) groups were significantly higher than those in the control group (0.19 and 0.73), whereas no significant differences were detected between the 2‰ (0.22 and 0.95) or 4‰ (0.36 and 2.08) groups and the control (*p* < 0.05) (Figure 3D,M). Crustin mRNA levels in the control group (0.74) did not differ significantly from those in the 4‰ (0.84) and 6‰ (1.47) groups but were significantly lower than those in the 2‰ (1.62) and 8‰ (3.91) groups (*p* < 0.05) (Figure 3K). mRNA levels of *Cpa2*, *TLR13*, and *CTLs* in the 8‰ group (0.54, 1.26, and 1.41) were significantly higher than those in the control group (0.38, 0.65, and 1.00), whereas no significant differences were observed between the 2‰ (0.51, 0.95, and 1.17), 4‰ (0.46, 0.70, and 1.08), or 6‰ (0.44, 0.77, and 1.09) groups and the control (*p* < 0.05) (Figure 3N–P). Collectively, these findings indicate that BPP induces adaptive modulation of immune function in *C. quadricarinatus*—encompassing physiological stress responses, antioxidant capacity, antibacterial activity, and anti-inflammatory capacity—in a concentration-dependent manner.

### 3.4. Transcriptome Sequencing of the Hepatopancreas of C. quadricarinatus Following Feeding with BPP

Transcriptomic profiling was conducted on hepatopancreas samples from *C. quadricarinatus* across different BPP treatment groups to elucidate the immunoregulatory mechanisms of BPP. A total of 44.21 GB of sequencing data were generated from 15 biological replicates. Before quality filtering, the mean values for total raw reads, total raw bases, Q20 percentage (percentage of nucleotides with mass value ≥ 20), Q30 percentage (percentage of nucleotides with mass value ≥ 30), and GC content were 46,615,831 reads, 6,992,374,700 bp, 99.17%, 98.35%, and 38.90%, respectively (Table S3). After filtering, the corresponding mean values were 46,602,012 reads, 6,927,475,161 bp, 99.18%, 98.36%, and 38.78%, respectively (Table S3). All samples achieved an alignment rate of 89% or higher with the *C. quadricarinatus* reference genome (NCBI accession number: GCF_026875155.1) (Table S3), indicating that the sample sources are reliable and that the sequencing quality of the samples is high. The correlation coefficients for biological replicates within each group exceeded 0.93 (Figure S1), indicating good reproducibility among samples within each group. Within the transcriptomic dataset, annotated known genes (reference genome genes) predominated, whereas novel genes were rare; the proportion of genes detected via sequencing represented 85.71% of all annotated genes in the reference genome (Table S4). These findings confirm the high quality of the transcriptome sequencing data.

### 3.5. Identification of DEGs

**Figure 4:**
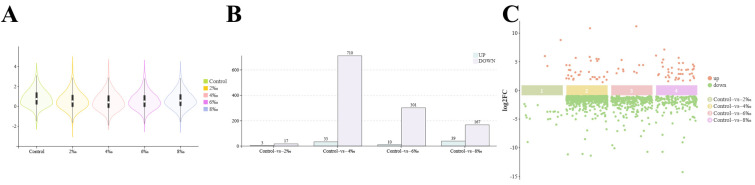
Identification of DEGs. (**A**) Visualization of gene expression levels in *Cherax quadricarinatus* across different BPP concentration groups. (**B**) The number of DEGs identified in *C. quadricarinatus* following treatment with BPP (2‰, 4‰, 6‰, and 8‰). (**C**) Log_2_(fold change) distribution of DEGs under BPP treatment conditions (2‰, 4‰, 6‰, and 8‰).

Gene expression was analyzed in hepatopancreas samples from *C. quadricarinatus* across different BPP treatment groups. All groups exhibited high and stable gene expression levels (Table S5; Figure 4A). Pairwise comparisons between the control and each treatment group—Control vs. 2‰, Control vs. 4‰, Control vs. 6‰, and Control vs. 8‰—yielded 20 (3 upregulated, 17 downregulated), 743 (33 upregulated, 710 downregulated), 311 (10 upregulated, 301 downregulated), and 206 (39 upregulated, 167 downregulated) DEGs, respectively (Table S5) (Figure 4B). Across all treatment groups, a total of 957 DEGs were identified, comprising 70 upregulated and 887 downregulated genes (Table S5; Figure 4B). Figure 4C illustrates the distribution of log_2_(fold change) values for all DEGs across the 2‰, 4‰, 6‰, and 8‰ treatment groups. These findings suggest that the 957 DEGs identified under the different BPP concentrations may represent regulatory genes involved in modulating immune function in *C. quadricarinatus*; however, this hypothesis requires further experimental validation.

### 3.6. GO and KEGG Enrichment Analyses and STEM Analysis of DEGs

**Figure 5:**
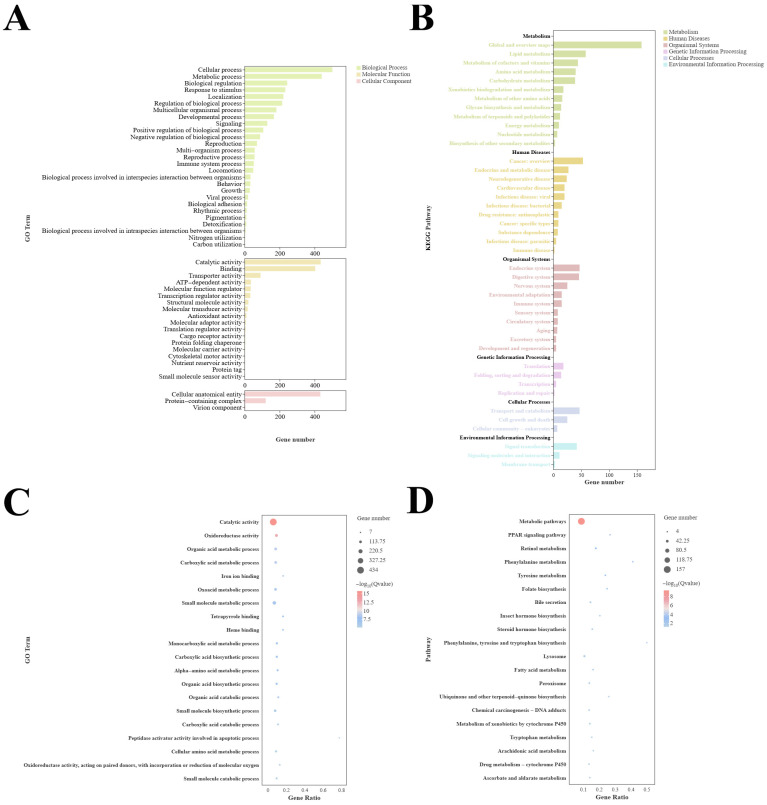
GO and KEGG enrichment analysis of DEGs. (**A**) Distribution of GO terms among the 957 DEGs. (**B**) Distribution of KEGG pathways among the 957 DEGs. (**C**) Significant enrichment of the top 20 GO terms among the 957 DEGs. (**D**) Significant enrichment of the top 20 KEGG pathways among the 957 DEGs.

**Figure 6:**
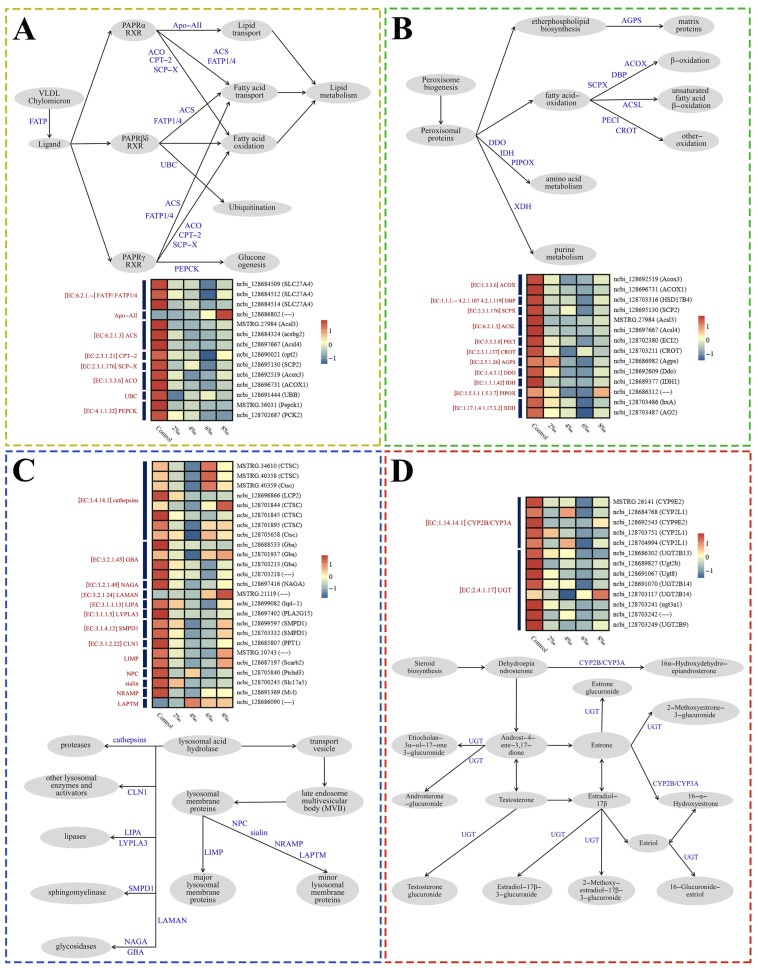
Four Simplified Pathway Diagrams. (**A**) represents the PPAR signaling pathway, (**B**) represents peroxisomes, (**C**) represents lysosomes, and (**D**) represents steroid hormone biosynthesis. The heatmaps show the specific distribution of these DEG types and their expression changes.

**Figure 7:**
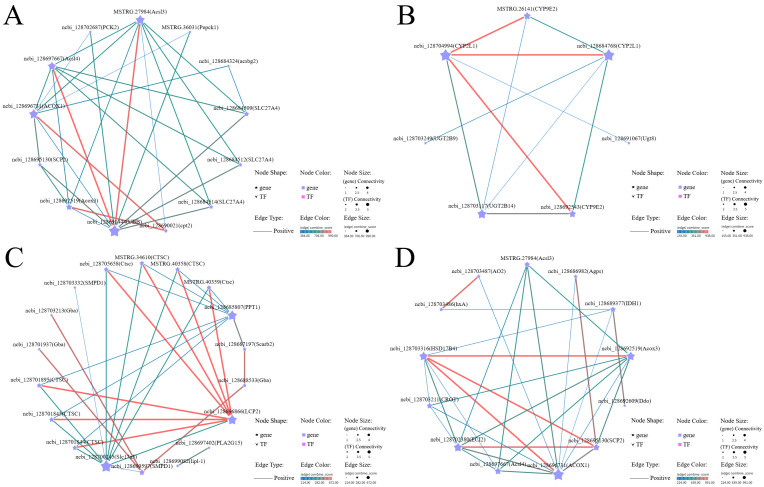
Visualized regulatory networks of the PPAR signaling pathway, peroxisomes, steroid hormone biosynthesis, and lysosomes. (**A**) Visualized regulatory network of the PPAR signaling pathway. (**B**) Visualized regulatory network of steroid hormone biosynthesis. (**C**) Visualized regulatory network of lysosomes. (**D**) Visualized regulatory network of peroxisomes.

To comprehensively elucidate the biological roles of the aforementioned DEGs, GO and KEGG enrichment analyses were performed. The 957 DEGs were annotated with 48 level II GO terms, spanning the three primary GO categories: Molecular Function, Biological Process, and Cellular Component (Table S6 and Figure 5A). Notably, the Biological Process category encompassed the highest number of DEGs, whereas the Cellular Component category contained the fewest (Table S6 and Figure 5A). Similarly, these DEGs were mapped to 287 level II KEGG pathways, which were classified into six major KEGG categories: Cellular Processes, Environmental Information Processing, Genetic Information Processing, Human Diseases, Metabolism, and Organismal Systems (Table S7 and Figure 5B). The Metabolism category harbored the largest proportion of DEGs, while Genetic Information Processing contained the smallest (Table S7 and Figure 5B). Subsequently, 214 GO terms and 20 KEGG pathways were identified as significantly enriched (*p* < 0.05) (Tables S8 and S9 and Figure 5C,D). In conjunction with relevant literature, we screened these to identify four GO terms and KEGG pathways related to immune function (physiological stress, antioxidant capacity, antibacterial activity, and anti-inflammatory capacity): the PPAR signaling pathway, peroxisome, steroid hormone biosynthesis, and lysosome (Table 1 and Figure 6). See Figure S2 for a visualization of the complete pathway map. Intriguingly, peroxisome (GO:0005777/ko04146) and lysosome (GO:0005764/ko04142) appeared in both annotation systems, implying that these organelles may play pivotal roles in the immune regulation of *C. quadricarinatus*. Within these selected terms and pathways, 14, 14, 13, and 25 DEGs were differentially regulated, respectively (Table 1 and Figure 6). Interaction network analysis revealed distinct regulatory architectures for these DEGs within the context of the PPAR signaling pathway, peroxisome, steroid hormone biosynthesis, and lysosome, with core gene clusters comprising 13, 13, 7, and 18 members, respectively (Table 1 and Figure 7A–D). These networks delineate the interaction patterns among DEGs, suggesting that the identified core genes are likely key mediators of BPP-mediated immune modulation in *C. quadricarinatus*. From these core networks, 14 core candidate genes implicated in immune regulation were extracted, and their distribution across the pathways is detailed in Table 1. STEM analysis identified two significant expression profiles, and the 14 previously identified core candidate genes were also found to be clustered within them (Table S10 and Figure S3). Cluster analysis showed that these 14 core candidate genes could be divided into two categories: the control group and the 2‰, 4‰, 6‰, and 8‰ groups (i.e., the 2–8‰ groups), with the control group exhibiting significantly higher gene expression levels (Table S11 and Figure S4). Future investigations will prioritize these four candidate pathways/terms and related regulatory networks and core candidate genes to validate their functional significance.

### 3.7. RT-qRCR

**Figure 8:**
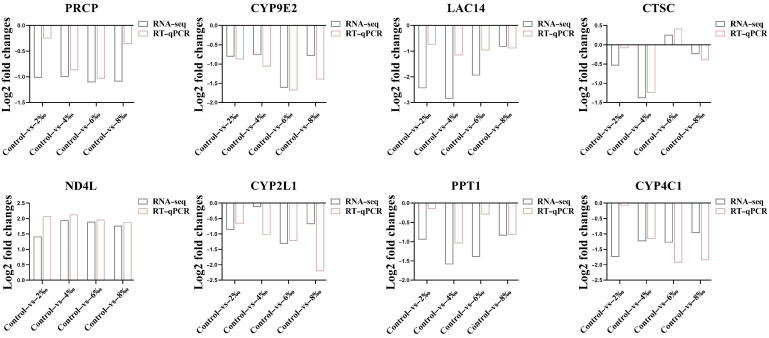
RT-qPCR Validation.

To validate the reliability of the RNA sequencing data, the expression levels of eight genes were randomly analyzed via RT-qPCR. These genes included lysosomal Pro-X carboxypeptidase-like (*PRCP*, MSTRG-11787), cytochrome P450 9e2-like (*CYP9E2*, MSTRG-26141), laccase (*LAC14*, MSTRG-31851), dipeptidyl peptidase 1-like (*CTSC*, MSTRG-34610), NADH dehydrogenase subunit 4L (*ND4L*, MSTRG-39988), cytochrome P450 2L1-like (*CYP2L1*, ncbi-128684768), palmitoyl-protein thioesterase 1-like (*PPT1*, ncbi-128685807), and cytochrome P450 4C1-like (*CYP4C1*, ncbi-128686658). The qRT-PCR results demonstrated that the expression profiles of these genes were consistent with the trends identified by RNA-seq analysis (Figure 8). This corroboration confirms the high validity of the transcriptomic data obtained from RNA-seq.

## 4. Discussion

The development of cost-effective feed additives that promote growth performance and possess immunomodulatory properties is essential for advancing the commercial aquaculture industry [48]. Accumulating evidence indicates that specific polysaccharides can significantly enhance the growth performance—measured as body length and weight—of various crustaceans. For instance, *Procambarus clarkii* [49], *Fenneropenaeus merguiensis* [50], and *Litopenaeus vannamei* [48] exhibited marked improvements in somatic measurements following administration of *Rhodiola rosea*, *Enteromorpha*, and *Astragalus membranaceus* polysaccharides, respectively. Similar to these observations, the present study demonstrated that *C. quadricarinatus* fed a high concentration (8‰) of BPP exhibited significant increases in both body length and weight. However, it is noteworthy that the efficacy of polysaccharides is not universal, as certain formulations, such as Yu-Ping-Feng polysaccharides, exert negligible effects on growth metrics [51]. Beyond growth promotion, polysaccharides serve multifunctional roles in crustaceans and are widely utilized as immunostimulants [52]. Recent investigations into crustacean immunity have predominantly focused on the hepatopancreas of species such as *Litopenaeus vannamei* [53], *Marsupenaeus japonicus* [54], *Eriocheir sinensis* [55], and *Portunus trituberculatus* [56]. Current data suggest that plant or animal polysaccharides can effectively facilitate hepatopancreatic tissue repair. For example, *Salvia miltiorrhiza* polysaccharide has been shown to mitigate hepatopancreatic tissue damage in *Procambarus clarkii*, thereby preserving organ integrity and function [57]; similarly, *Cipangopaludina chinensis* polysaccharides ameliorate hepatic injury in murine models [11]. Similarly to these findings, dietary supplementation with BPP in this study resulted in the repair of mild hepatopancreatic tissue damage in *C. quadricarinatus*, with the most pronounced restorative effect observed at the highest concentration (8‰). The immune function of crustaceans encompasses physiological stress responses, antioxidant capacity, antibacterial activity, and anti-inflammatory processes. *Agaricus bisporus* polysaccharides exhibit potent antibacterial and anti-inflammatory properties, effectively counteracting *Aeromonas salmonicida* infection and intestinal inflammation in *Procambarus clarkii* [58]. Furthermore, long-term administration of trehalose and galactooligosaccharides has been shown to significantly enhance the antioxidant and stress resistance capabilities of *C. quadricarinatus* and *Litopenaeus vannamei* [3,59]. Collectively, these findings suggest that the hepatoprotective effect of BPP observed in this study is likely mediated through the modulation of immune function in *C. quadricarinatus*.

This study provides a comprehensive analysis of alterations in the immune function of *C. quadricarinatus* following BPP ingestion. *Hsp70* and *hsp90* are ATP-dependent molecular chaperones essential for protein quality control, signal transduction, and stress adaptation [27]. Concurrently, increased phosphatase activity influences phosphorylation–hydrolysis homeostasis, potentially mitigating the accumulation of metabolic waste [25]; this mechanism may be intrinsically linked to the roles of ACP and AKP in stress signaling and physiological adaptation [28]. In this study, compared with the control group, the activity of ACP and AKP, along with the transcriptional levels of *hsp70* and *hsp90*, was significantly elevated in the high-concentration (6‰ and 8‰) treatment groups (Figure 3A–D). We speculate that ingestion of high concentrations of BPP (6‰ and 8‰) attenuates physiological stress in *C. quadricarinatus*, thereby enhancing the environmental adaptability of the hepatopancreatic tissue. Furthermore, following BPP administration, the activities of CAT, SOD, and GSH-Px were significantly upregulated, whereas MDA levels were markedly reduced (Figure 3E–H). The synergistic action of these antioxidant enzymes mitigates toxicity arising from superoxide anion radicals [29,30]. Specifically, CAT and GSH-Px catalyze the decomposition of hydrogen peroxide into non-toxic substances, while SOD scavenges reactive oxygen species (ROS), thereby alleviating lipid peroxidation [31,32]. As a byproduct of lipid peroxidation that induces tissue damage, MDA serves as a critical biomarker for assessing the extent of oxidative stress and cellular damage [33]. The enhancement of antioxidant capacity directly correlates with a significant reduction in oxidative injury [60]. Consequently, we hypothesize that dietary BPP supplementation bolsters the antioxidant defense system of *C. quadricarinatus*, protecting hepatopancreatic tissue from oxidative damage. Data indicate that invertebrates rely heavily on antimicrobial peptides (AMPs) and other innate immune factors to combat invading pathogens, given the absence of an adaptive immune system [61,62]. AMPs function as key mediators of innate immunity by disrupting microbial pathogens, including bacteria, fungi, and viruses [63,64]. Both *ALF* and crustin are families of AMPs exhibiting broad-spectrum antimicrobial activity against Gram-positive bacteria, Gram-negative bacteria, and fungi [34,35]. *ALFs* neutralize bacterial lipopolysaccharides (LPS), while crustins induce bacterial membrane leakage and structural disintegration [65,66]. LYS, a canonical AMP, exerts its bactericidal effect by hydrolyzing bacterial cell wall peptidoglycan [36]. Additionally, the recognition of microbial polysaccharides by pattern recognition receptors (PRRs) triggers the *proPO* cascade, leading to melanin synthesis and deposition on pathogen surfaces [67]. The highly toxic quinone compounds and ROS intermediates generated during *proPO* activation directly contribute to the elimination of invading microorganisms [68,69,70]. Therefore, *PO* and *proPO* are established immunological markers for evaluating antibacterial activity [37]. In this study, compared with the control group, LYS activity and the gene expression of *ALF*, crustin, *PO*, and *proPO* were significantly increased in the high-concentration (8‰) treatment group (Figure 3I–M). We hypothesize that ingestion of 8‰ BPP enhances antimicrobial defenses, fortifying the pathogen resistance of the hepatopancreatic tissue. Regarding inflammatory responses, *TLR13* serves as a crucial indicator for evaluating inflammatory damage [38,39]. *Cpa2* exhibits anti-inflammatory effects by cleaving pro-inflammatory mediators, while *CTLs* modulate the equilibrium between inflammatory and anti-inflammatory pathways [40,41]. In this study, the gene expression of *TLR13*, *Cpa2*, and *CTLs* was significantly upregulated in the 8‰ treatment group compared to the control (Figure 3N–P). We speculate that consumption of 8‰ BPP enhances the anti-inflammatory capacity of *C. quadricarinatus*, thereby mitigating inflammatory damage in the hepatopancreas.

Subsequently, this study elucidates alterations in immune function based on transcriptomic profiling. Candidate GO terms, KEGG pathways, and gene functions were identified as potentially relevant to the immune regulation induced by BPP ingestion in *C. quadricarinatus*. Polysaccharides are typically recognized by pattern recognition receptors (PRRs) on immune cells, thereby activating downstream immune signaling pathways [71]. The Peroxisome Proliferator-Activated Receptor (PPAR) signaling pathway plays a pivotal role in modulating anti-inflammatory responses. PPARs, a family of nuclear receptors governing cellular energy metabolism, can antagonize the activation and function of *NF-κB* during inflammatory responses, thereby exerting anti-inflammatory effects [72,73]. The current literature indicates that all three PPAR isoforms (*PPARα*, *PPARβ/δ*, and *PPARγ*) possess distinct anti-inflammatory properties [74,75]. Furthermore, the PPAR signaling pathway regulates inflammatory homeostasis by orchestrating fatty acid transport and binding [76,77]. In this study, *SLC27A4*, *Acsl3*, *SCP2*, *Acox3*, and *ACOX1* exhibited high expression in the control group and low expression in the 2–8‰ groups, demonstrating an overall downward trajectory (Figure 6). *SLC27A4* encodes a fatty acyl-CoA synthetase that activates very-long-chain fatty acids into their corresponding CoA derivatives [78]. Notably, ferroptosis—an iron-dependent form of regulated cell death—is intricately linked to inflammatory processes [79]. Inhibition of *SLC27A4* sensitizes cells to ferroptosis, thereby influencing inflammation [80]. Similarly, *Acsl3* encodes a key isoform implicated in ferroptosis and plays a crucial role in driving inflammatory progression [81]. Conversely, inhibition of *Acsl3* prevents ferroptosis in macrophages, thereby mitigating organ fibrosis and preserving an anti-inflammatory microenvironment [82]. Moreover, knockdown of *Acsl3* attenuates the production of pro-inflammatory cytokines (e.g., *TNF-α*), effectively suppressing inflammatory responses [83]. In contrast, *SCP2* facilitates the activation of type II natural killer T cells to produce pro-inflammatory cytokines, thereby exacerbating inflammation [84,85]. *Acox3* is likely involved in tumor-associated chronic inflammation and immune regulatory networks [86]. In *Larimichthys crocea*, suppression of *Acox1* expression correspondingly reduces the transcription of pro-inflammatory genes such as *IL-6* [87]. We hypothesize that the proteins encoded by these genes possibly modulate inflammatory responses in tissues (e.g., the hepatopancreas), thereby influencing the immune defense status of *C. quadricarinatus*.

Steroid hormone biosynthesis is intrinsically linked to physiological stress responses. Steroid hormones govern a wide array of physiological processes, encompassing osmoregulation, sexual maturation, reproduction, and stress adaptation [88]. Previous investigations have established steroid hormone biosynthesis as a critical mediator of ammonia-induced stress adaptation in *Litopenaeus vannamei*, as well as a key component in its response to salinity stress [89,90]. In this study, *CYP9E2*, *CYP2L1*, and *UGT2B14* within the steroid hormone biosynthesis pathway exhibited high expression in the control group and low expression in the 2–8‰ groups, demonstrating an overall downward trajectory (Figure 6). Cytochrome P450 monooxygenases constitute a pivotal enzyme system responsible for the metabolism of endogenous and exogenous compounds, playing a significant role in the pathogenesis of inflammatory dysregulation and stress responses [91,92]. For instance, under heavy metal (e.g., lead) and pesticide (e.g., Spinosad) stress, *CYP9E2* expression was significantly upregulated in *Procambarus clarkii* and *Leptinotarsa decemlineata* [93,94]. Similarly, *CYP2L1* expression undergoes significant modulation during salinity- and mercury-induced stress in *Procambarus clarkii* and *Litopenaeus vannamei* [95,96]. Furthermore, UDP-glycosyltransferase (UGT) catalyzes the transfer of glycosyl moieties (e.g., glucuronic acid) to xenobiotic or endogenous substrates, facilitating detoxification through the modification of metabolites [97,98]. Consistent with this function, *UGT2B14* expression in *Macrobrachium rosenbergii* was significantly upregulated under Decapod iridescent virus 1-induced stress [99]. We speculate that these genes modulate physiological stress in *C. quadricarinatus* during BPP ingestion by orchestrating steroid hormone biosynthesis.

Lysosomes and peroxisomes are pivotal organelles orchestrating autophagic processes [100]. Autophagy, an evolutionarily conserved catabolic mechanism, is widely recognized as a survival strategy that confers antioxidant and antimicrobial defense [101]. Currently, the protein degradation machinery of autophagy is extensively leveraged to combat bacterial infections at multiple regulatory levels, establishing a functional nexus between autophagy and antimicrobial immunity [102]. Specifically, the activation of autophagy can bidirectionally modulate responses to pathogen invasion by either promoting or suppressing antimicrobial immunity [103]. In this study, *Gba*, *CTSC*, *PPT1*, and *lipl-1* within the lysosome pathway exhibited high expression in the control group and low expression in the 2–8‰ groups, demonstrating an overall downward trajectory (Figure 6). *Gba*, located on the lysosomal membrane, hydrolyzes membrane glycoproteins into ceramides and glucose [104]. Dysfunction of *Gba* impairs phagosome-lysosome fusion and disrupts autophagic flux, thereby preventing the effective translocation of pathogens into a degradative milieu [105,106,107]. In invertebrates, cysteine proteases constitute a major component of the lysosomal proteolytic system [108]. *CTSC*, a lysosomal cysteine protease, plays a crucial role in anti-inflammatory, antibacterial, and antiviral immunity [109,110]. For instance, in *Sinonovacula constricta*, *Eriocheir sinensis*, and *Marsupenaeus japonicus* challenged with specific pathogens (e.g., *Vibrio anguillarum*, *Vibrio alginolyticus*, and *White Spot Syndrome Virus*), *CTSC* expression is significantly changed [111,112,113]. *PPT1*, a lysosomal hydrolase, catalyzes the cleavage of thioester bonds in palmitoylated substrates, thereby facilitating their degradation [114,115]. Knockdown of *PPT1* results in the accumulation of dysfunctional lysosomes and lipoylated proteins, influencing the role of autophagy in pathogen clearance [116]. Furthermore, the antibacterial activity of *lipl-1* is associated with lipid hydrolysis [117]. In lipid-rich environments, *lipl-1* significantly affects the growth of bacteria such as *Escherichia coli*, *Salmonella* spp., and *Legionella* spp. [118]. We hypothesize that upon ingestion of BPP, the proteins encoded by these genes impact the degradation of pathogens by regulating lysosome-related processes in *C. quadricarinatus*.

Autophagy is intrinsically linked to oxidative stress. Specifically, while oxidative stress can induce autophagic flux, autophagy mitigates oxidative damage through the sequestration and degradation of reactive oxygen species (ROS) and the turnover of peroxisomes [119,120]. In this study, the concurrent identification of peroxisomes and lysosomes within the autophagy process in both GO terms and KEGG pathways underscores the pivotal regulatory role of BPP in modulating the immune function of *C. quadricarinatu*. Furthermore, peroxisomes are critical organelles governing ROS production, redox signaling, β-oxidation of fatty acids, and lipid homeostasis [121,122]. They harbor various oxidases—such as flavin oxidase, uric acid oxidase (UoX), and acetyl-CoA oxidase—capable of generating hydrogen peroxide [123]. Concomitantly, peroxisomal catalase and other antioxidant enzymes degrade toxic superoxide radicals into innocuous byproducts, thereby maintaining intracellular redox homeostasis [124,125]. In this study, *ACOX1*, *Acox3*, *IDH1*, *Ddo*, and *SCP2* within the peroxisome pathway exhibited high expression in the control group and low expression in the 2–8‰ groups, demonstrating an overall downward trajectory (Figure 6). *ACOX1* encodes a key rate-limiting enzyme in the β-oxidation pathway [126,127]. In *Larimichthys crocea*, suppression of *ACOX1* expression results in reduced ROS levels and upregulation of antioxidant-related genes, such as catalase [87]. Both *ACOX1* and *Acox3* generate hydrogen peroxide as a byproduct during fatty acid oxidation catalysis [127,128]; consequently, as a highly reactive oxidant, hydrogen peroxide can induce oxidative damage [129]. For instance, in *Procambarus clarkii*, upregulation of *Acox3* elevates ROS and hydrogen peroxide production, precipitating oxidative damage [95]. *IDH1* plays a pivotal role in regulating redox balance and defending against oxidative damage by modulating glucose utilization via the pentose phosphate pathway [130,131]; notably, during copper stress in *Procambarus clarkii*, *IDH1* assumes a significant role in the antioxidant system [132]. *Ddo* generates hydrogen peroxide during D-aspartate catabolism and exerts pro-oxidant effects [133]; however, it indirectly shields cells from severe oxidative damage by regulating D-aspartate levels [134]. *SCP2*, also known as nonspecific lipid transfer protein, is vital for intracellular lipid transport and metabolism (e.g., phospholipids, fatty acids, and cholesterol) [135]. Inhibition of *SCP2* significantly attenuates ROS accumulation, thereby protecting cells from oxidative damage [136]. We hypothesize that upon ingestion of BPP, the proteins encoded by these genes, by participating in peroxisome-related processes, thus exert antioxidant functions. It is noteworthy that *SCP2*, *Acox3*, and *Acox1* were identified as common genes in two enriched KEGG pathways (Peroxisome and PPAR signaling pathway) (Table 1). We speculate that the expression of these genes may coordinately regulate both pathways, integrating antioxidant functions with anti-inflammatory capabilities; however, this crosstalk warrants further investigation.

**Figure 9:**
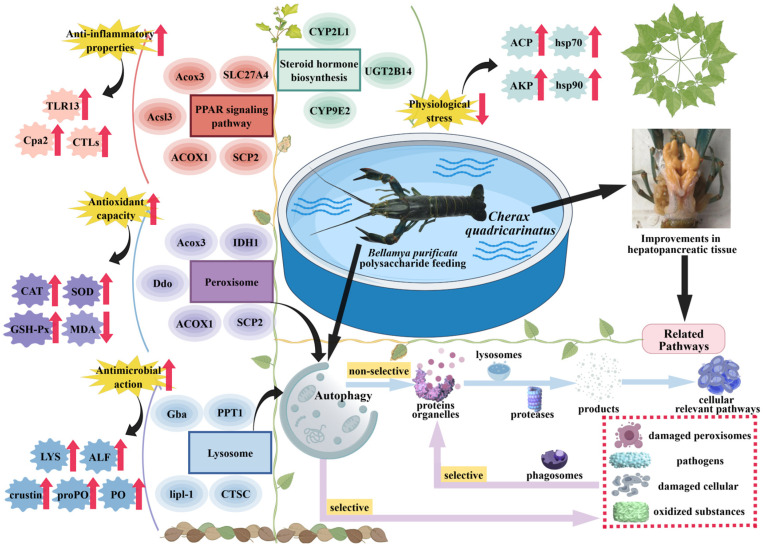
Schematic overview of the mechanism by which BPP regulates immune function in *Cherax quadricarinatus*. The four differently colored rectangles, connected by relevant pathways, represent the four candidate pathways involved. The 14 differently colored oval boxes surrounding the rectangles represent the 14 candidate genes within these pathways. The purple and blue arrows below the pool and their associated symbols represent the autophagy process. The yellow star-shaped boxes represent the immune functions corresponding to the pathways and genes, while the red upward and downward arrows represent increases and decreases, respectively. The star-shaped boxes of various colors (other than yellow) represent the indicators (enzyme activity or gene expression) corresponding to the immune functions.

Based on the findings of this study, a mechanistic model was constructed to elucidate the regulatory mechanisms by which BPP modulates the immune function of *C. quadricarinatus* (Figure 9). Dietary supplementation with BPP alleviated hepatopancreatic tissue damage, thereby orchestrating the activation of multiple signaling pathways and genes. Specifically, pathways such as the PPAR signaling pathway and steroid hormone biosynthesis, along with key genes including *Acsl3* and *CYP2L1*—which are intrinsically linked to physiological stress responses and anti-inflammatory capacity—were activated. This activation led to attenuated physiological stress and enhanced anti-inflammatory capacity in *C. quadricarinatus*, subsequently modulating corresponding immune biomarkers (enzyme activity or gene expression). Autophagy serves as a critical homeostatic process for clearing intracellular pathogens, damaged organelles, and misfolded proteins [137]. Administration of BPP appears to activate autophagic flux, facilitating the extensive, non-selective degradation of intracellular components via lysosomes and associated proteases (e.g., *CTSC*) [24]. In this context, non-selective autophagy involves the indiscriminate engulfment and degradation of cytoplasmic constituents, such as intracellular bacteria and host proteins [137]; consequently, lysosomes, as the terminal degradative compartment of this process, play a decisive role in determining the fate of engulfed pathogens [138]. Subsequently, the resulting degradation products are recycled into relevant metabolic pathways, thereby sustaining cellular metabolic homeostasis [100]. In contrast, selective autophagy targets specific cellular components for degradation [139]. Consequently, damaged organelles, invading pathogens, oxidized biomolecules, and dysfunctional peroxisomes are selectively sequestered and cleared, thereby regulating protein and organelle quality control [103,119,140]. Pathways associated with antimicrobial activity and antioxidant capacity (e.g., lysosomes and peroxisomes) and genes (e.g., *PPT1* and *ACOX1*) play pivotal roles in this regulatory network. They collectively contribute to enhanced antimicrobial defense and antioxidant capacity, thereby modulating relevant immune markers (enzyme activity or gene expression). In summary, these coordinated genetic and pathway alterations enable *C. quadricarinatus* to adapt to BPP-containing diets through a sophisticated mechanism involving the dynamic regulation of immune function.

## 5. Conclusions

This study demonstrated that dietary supplementation with BPP significantly improved the growth performance of *C. quadricarinatus* and mitigated hepatopancreatic tissue damage, as evidenced by the attenuation of stellate tubule constriction and vacuolization. Furthermore, the addition of BPP to the diet also had positive effects on immune functions, including physiological stress response, antioxidant capacity, antimicrobial activity, and anti-inflammatory capacity, as reflected in changes in corresponding enzyme activities and gene expression. Through integrated transcriptomic analysis, this study identified critical GO terms (e.g., Peroxisome and Lysosome), KEGG pathways (e.g., PPAR signaling pathway and Steroid hormone biosynthesis), and candidate genes (e.g., *Acox3* and *ACOX1*) associated with the immune regulation of *C. quadricarinatus*. Based on these data, a mechanistic model delineating the potential regulatory network underlying the response to BPP supplementation was constructed. These findings provide a scientific foundation for developing novel disease prevention strategies in *C. quadricarinatus* aquaculture and lay the groundwork for the future application of BPP as a functional additive in aquatic animal feeds.

## Figures and Tables

**Table 1:** Candidate genes identified in the four candidate GO terms and KEGG pathways of *Cherax quadricarinatus*.

Candidate GO Term or KEGG Pathways	ID	*p* Value	Q Value	Background Number	Core Genes Number	Candidate Genes Number	Candidate Genes ID	Candidate Genes
PPAR signaling pathway	ko03320	4.4947 × 10^−7^	0.000064	14	13	5	MSTRG.27984 ncbi_128684514 ncbi_128692519 ncbi_128695130 ncbi_128696731	long-chain-fatty-acid–CoA ligase 4-like (*Acsl3*) long-chain fatty acid transport protein 4-like (*SLC27A4*) peroxisomal acyl-coenzyme A oxidase 3-like (*Acox3*) sterol carrier protein 2-like (*SCP2*) peroxisomal acyl-coenzyme A oxidase 1-like (*ACOX1*)
Peroxisome	ko04146/GO:0005777	0.001125/0.000420	0.023734/0.038530	14	13	5	ncbi_128689377 ncbi_128692519 ncbi_128692609 ncbi_128695130 ncbi_128696731	isocitrate dehydrogenase [NADP] cytoplasmic-like (*IDH1*) peroxisomal acyl-coenzyme A oxidase 3-like (*Acox3*) D-aspartate oxidase-like (*Ddo*) sterol carrier protein 2-like (*SCP2*) peroxisomal acyl-coenzyme A oxidase 1-like (*ACOX1*)
Steroid hormone biosynthesis	ko00140	0.000519	0.01497	13	7	3	ncbi_128684768 ncbi_128692543 ncbi_128703117	cytochrome P450 2L1-like (*CYP2L1*) cytochrome P450 9e2-like (*CYP9E2*) UDP-glycosyltransferase UGT5-like (*UGT2B14*)
Lysosome	ko04142/GO:0005764	0.000894/0.025560	0.023326/0.527499	25	18	4	ncbi_128685807 ncbi_128688533 ncbi_128699082 ncbi_128701845	Palmitoyl-protein thioesterase 1-like (*PPT1*) lysosomal acid glucosylceramidase-like (*Gba*) lipase 3-like (*lipl-1*) dipeptidyl peptidase 1-like (*CTSC*)

## Data Availability

The data presented in this study is openly available on NCBI. The registration number is PRJNA1467218.

## Supplementary Materials

The following supporting information can be downloaded at: https://www.mdpi.com/article/10.3390/antiox15070907/s1. Table S1: The composition of the experimental diets for *Cherax quadricarinatus* (‰ of dried diet); Table S2: Gene primer sequences; Table S3: Data statistics; Table S4: Statistics on Genotype Types; Table S5: Distribution of 957 significantly differentially expressed genes; Types; Table S6: Distribution of GO terms among DEGs; Table S7: Distribution of KEGG Pathways among DEGs; Table S8: Enrichment statistics of DEGs in GO term; Table S9: Enrichment statistics of DEGs in KEGG Pathway; Table S10: DEGs with significant expression profiles in STEM analysis (profiles 1); DEGs with significant expression profiles in STEM analysis (profiles 0); Table S11: 14 candidate genes associated with immune function in *Cherax quadricarinatus*; Figure S1: Sample correlation heatmap. Both the x-axis and y-axis represent individual samples, and the shade of colour indicates the magnitude of the correlation coefficient between two samples. The closer the colour is to red, the stronger the correlation; the closer it is to blue, the weaker the correlation; Figure S2: Visualized pathway diagrams for the PPAR signaling pathway, peroxisomes, steroid hormone biosynthesis, and lysosomes. The red boxes indicate the types of upregulated or downregulated DEGs, and the heatmaps show the specific distribution of these DEG types and their expression changes; Figure S3: Eight expression patterns of DEGs. Significant expression patterns are shown as colored subplots. The lower left corner displays the p-value, the top shows the number of DEGs, and the right side provides a legend for the 14 candidate genes; Figure S4: Gene expression heatmap and hierarchical clustering of the 14 candidate genes.
