# Supplementary material for: Comparative Transcriptomics Reveals Potential Regulatory Mechanisms Underlying Changes in Antioxidant, Anti-Inflammatory, and Other Immune Functions Induced by Bellamya purificata Polysaccharides in Cherax quadricarinatus
Source: Antioxidants (Basel). 2026 Jul 22;15(7):907. doi: 10.3390/antiox15070907 (PMC13403722; doi:10.3390/antiox15070907)
Supplement: Supplementary file 1 [file antioxidants-15-00907-s001.zip › Figure S1-S4.pdf]

Figure legends

Figure S1. Sample correlation heatmap. Both the x-axis and y-axis represent individual samples, and the shade of colour indicates the magnitude of the correlation coefficient between two samples. The closer the colour is to red, the stronger the correlation; the closer it is to blue, the weaker the correlation.

Figure S2. Visualized pathway diagrams for the PPAR signaling pathway, peroxisomes, steroid hormone biosynthesis, and lysosomes. The red boxes indicate the types of upregulated or downregulated DEGs, and the heatmaps show the specific distribution of these DEG types and their expression changes.

Figure S3. Eight expression patterns of DEGs. Significant expression patterns are shown as colored subplots. The lower left corner displays the p-value, the top shows the number of DEGs, and the right side provides a legend for the 14 candidate genes.

Figure S4. Gene expression heatmap and hierarchical clustering of the 14 candidate genes.

Figure S1

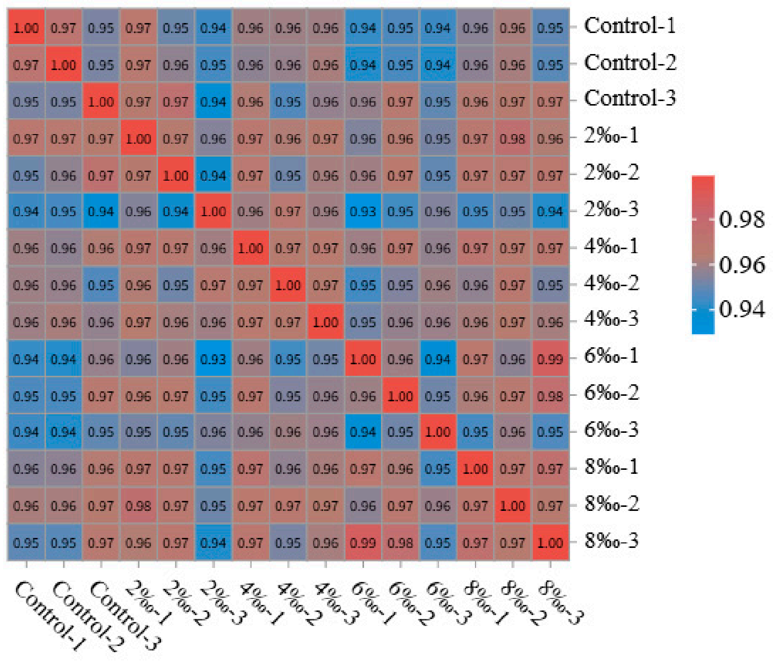
Control-1
Control-2
Control-3
2‰-1
2‰-2
2‰-3
4‰-1
4‰-2
4‰-3
6‰-1
6‰-2
6‰-3
8‰-1
8‰-2
8‰-3
0.98
0.96
0.94

Figure S2

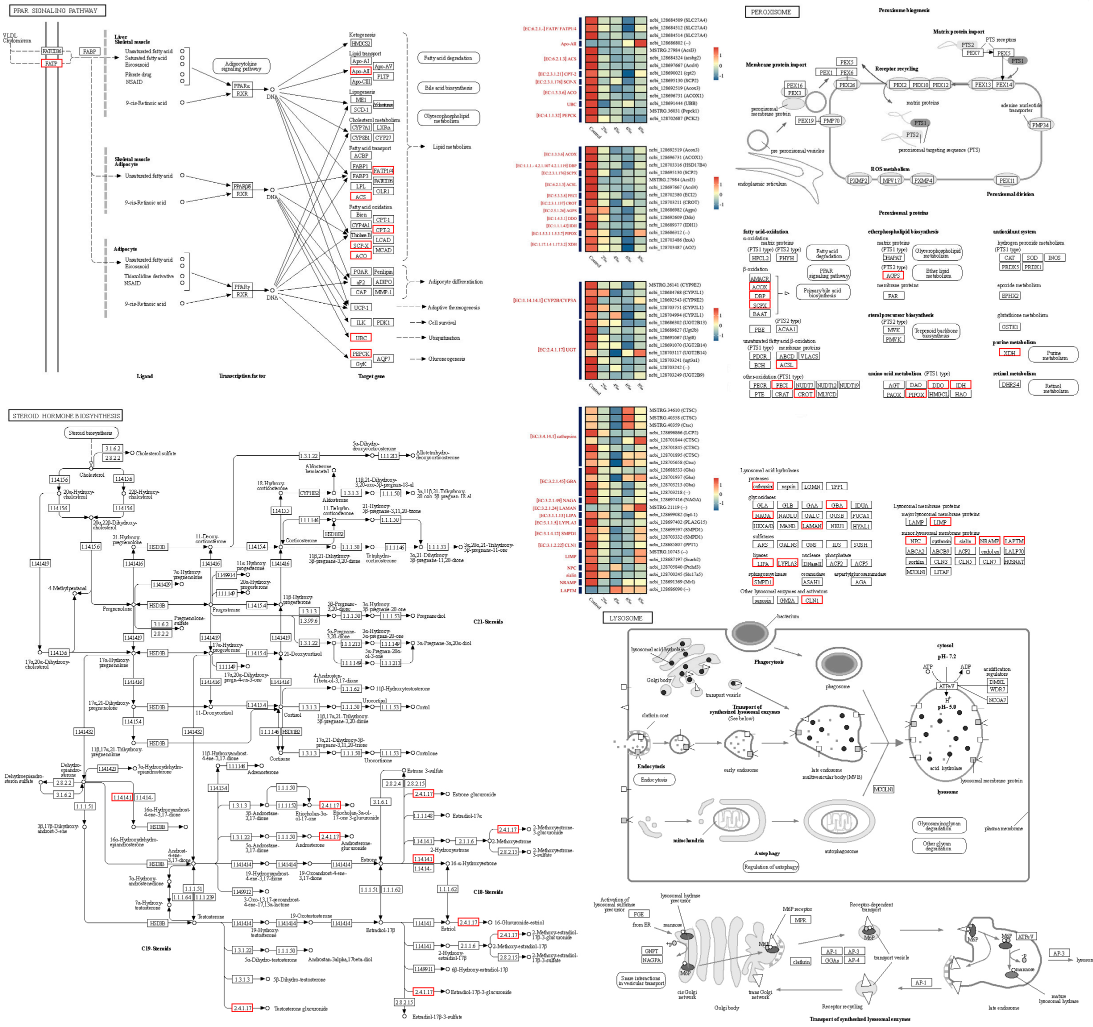
PPAR SIGNALING PATHWAY
PEROXISOME
STEROID HORMONE BIOSYNTHESIS
LYSOSOME

Figure S3

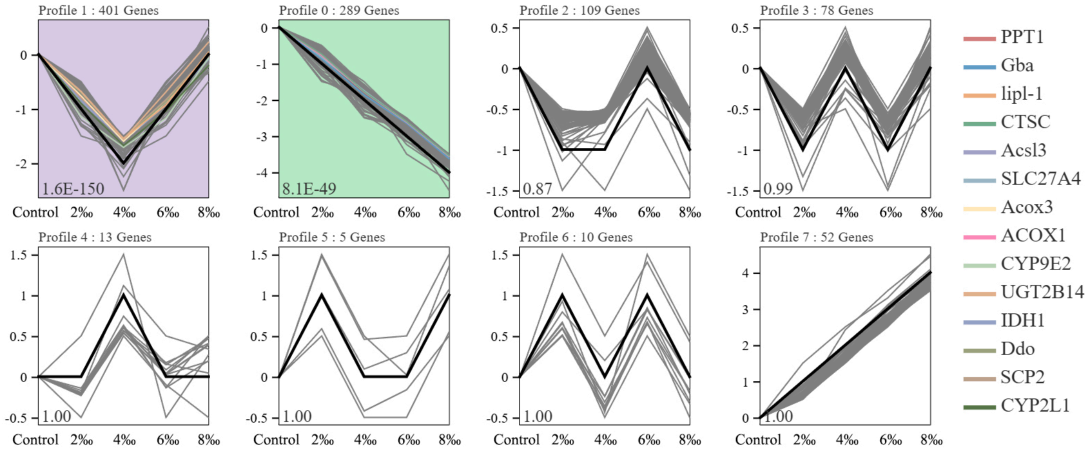
Profile 1 : 401 Genes
1.6E-150
Profile 0 : 289 Genes
8.1E-49
Profile 2 : 109 Genes
0.87
Profile 3 : 78 Genes
0.99
Profile 4 : 13 Genes
1.00
Profile 5 : 5 Genes
1.00
Profile 6 : 10 Genes
1.00
Profile 7 : 52 Genes
1.00
Control 2‰ 4‰ 6‰ 8‰
PPT1
Gba
lipl-1
CTSC
Acsl3
SLC27A4
Acox3
ACOX1
CYP9E2
UGT2B14
IDH1
Ddo
SCP2
CYP2L1

Figure S4

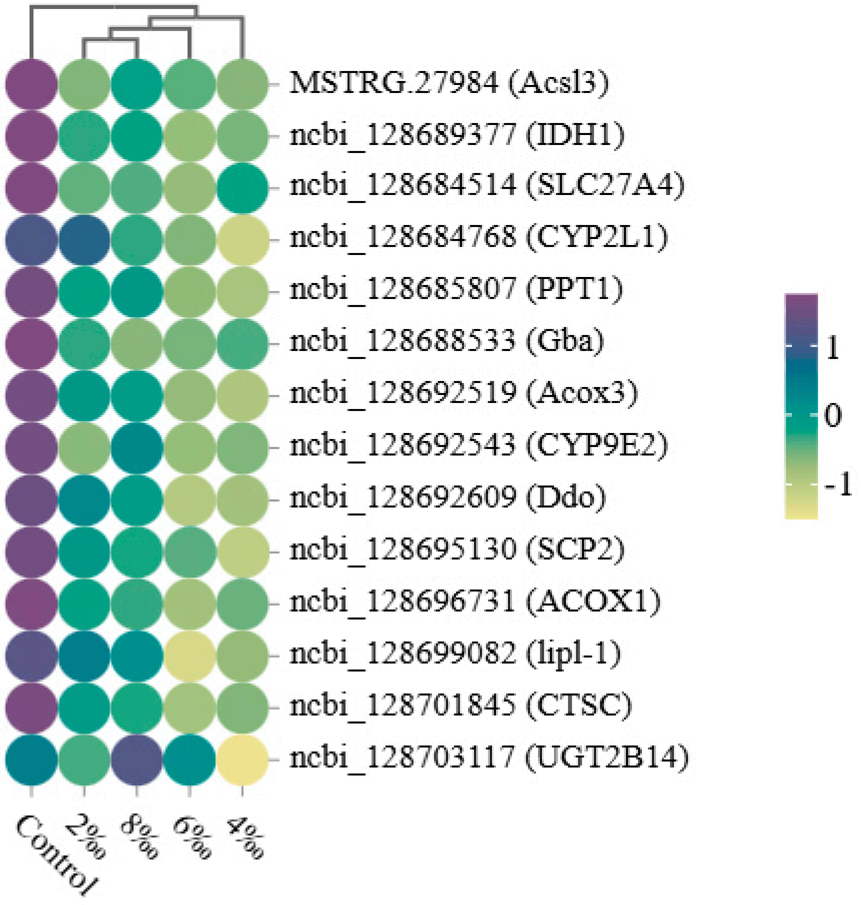
MSTRG.27984 (Acsl3)
ncbi_128689377 (IDH1)
ncbi_128684514 (SLC27A4)
ncbi_128684768 (CYP2L1)
ncbi_128685807 (PPT1)
ncbi_128688533 (Gba)
ncbi_128692519 (Acox3)
ncbi_128692543 (CYP9E2)
ncbi_128692609 (Ddo)
ncbi_128695130 (SCP2)
ncbi_128696731 (ACOX1)
ncbi_128699082 (lipl-1)
ncbi_128701845 (CTSC)
ncbi_128703117 (UGT2B14)
Control
2‰
8‰
6‰
4‰
1
0
-1
